# BAK1 Mediates Light Intensity to Phosphorylate and Activate Catalases to Regulate Plant Growth and Development

**DOI:** 10.3390/ijms21041437

**Published:** 2020-02-20

**Authors:** Shan Zhang, Cheng Li, Haihua Ren, Tong Zhao, Qi Li, Shufen Wang, Yanfeng Zhang, Fangming Xiao, Xiaofeng Wang

**Affiliations:** 1 State Key Laboratory of Crop Stress Biology for Arid Areas, College of Horticulture, Northwest A&F University, Yangling 712100, Shaanxi, China; Shan7842@vandals.uidaho.edu (S.Z.); lceye@nwafu.edu.cn (C.L.); rhh0530@nwafu.edu.cn (H.R.); ZT18829352848@gmail.com (T.Z.); lqi7@vt.edu (Q.L.); shufenwang@nwafu.edu.cn (S.W.);; 2 Department of Plant Sciences, University of Idaho, Moscow, ID 83844, USA; 3 Hybrid Rapeseed Research Center of Shaanxi Province, Yangling 712100, Shaanxi, China

**Keywords:** BAK1, high light, Catalase, receptor-like kinase, signal transduction

## Abstract

BAK1 (brassinosteroid-insensitive 1 (BRI1) associated receptor kinase 1) plays major roles in multiple signaling pathways as a coreceptor to regulate plant growth and development and stress response. However, the role of BAK1 in high light signaling is still poorly understood. Here we observed that overexpression of BAK1 in Arabidopsis interferes with the function of high light in promoting plant growth and development, which is independent of the brassinosteroid (BR) signaling pathway. Further investigation shows that high light enhances the phosphorylation of BAK1 and catalase activity, thereby reducing hydrogen peroxide (H_2_O_2_) accumulation. Catalase3 (CAT3) is identified as a BAK1-interacting protein by affinity purification and LC-MS/MS analysis. Biochemical analysis confirms that BAK1 interacts with and phosphorylates all three catalases (CAT1, CAT2, and CAT3) of *the* Arabidopsis genome, and the trans-phosphorylation sites of three catalases with BAK1-CD are identified by LC-MS/MS in vitro. Genetic analyses reveal that the BAK1 overexpression plants knocked out all the three *CAT* genes completely abolishing the effect of BAK1 on suppression of high light-promoted growth. This study first unravels the role of BAK1 in mediating high light-triggered activation of CATs, thereby degrading H_2_O_2_ and regulating plant growth and development in Arabidopsis.

## 1. Introduction

Light is one of the most prominent environmental factors regulating plant growth and development at multiple levels, including light quality, intensity, and duration [1,2]. In particular, changes in light intensity can cause dramatic changes in many plant physiological processes [3,4,5]. For example, Arabidopsis grown under different light intensities exhibits significant growth and developmental variations: Under strong light, plants grow rapidly accompanied by early bolting and flowering, suggesting high light can enhance the vegetative growth rate and accelerate reproductive transition [2,4,6].

Although it is well known that high light has a great influence on plant growth and development, the underlying precise molecular mechanisms are poorly understood. The early hypothesis suggests that Arabidopsis plants exhibit two separate responses in low light and high light [4]. The acclimation induced by high light renders higher rates of photosynthesis, leading to a higher rate of plant growth. In addition, since plants grown under high light become mature earlier, they usually also flower much earlier than plants grown in low light [4]. However, recent discoveries have implicated that the regulation of high light-dependent plant growth is more complicated and involves multiple cellular signaling pathways. For example, Feng et al. showed that in Arabidopsis, a chloroplast-derived signal is critical for high light-regulated flowering, which is mediated by the *FLOWERING LOCUS C* (*FLC*) gene, suggesting the chloroplast retrograde signal plays an important role in the high light-induced flowering [2].

Reactive oxygen species (ROS) have also been demonstrated to play multifaceted roles in plant growth and development. In general, ROS include the superoxide anion (O_2_^−^), hydrogen peroxide (H_2_O_2_), and hydroxyl radicals (OH·) [7]. ROS metabolism is tightly controlled by a variety of regulatory proteins involved in the redox process [8]. Cellular redox homeostasis is recognized as an integrator of information from different cellular signaling pathways, including metabolism and the environment controlling plant growth and acclimation responses, as well as cell suicide events [9]. For example, the redox states of components involved in photosynthesis, such as plastoquinone, show rapid and often transient shifts in response to changes in light [9]; Melatonin, an auxin-like indoleamine compound, can induce H_2_O_2_ generation in alfalfa seedlings, resulting in the induction of lateral roots [10]. And the H_2_O_2_ level significantly increases during bolting and flowering time in Arabidopsis [11].

Membrane receptor-like kinases (RLKs) play indispensable roles in the perception of external environment signals via transducing extracellular signals into intracellular signaling [12,13,14,15,16]. Among them, BAK1 (BRI1-associated Receptor Kinase 1) is a leucine-rich repeat (LRR) transmembrane RLK protein with a typical plant receptor kinase structure: five LRR repeats in the extracellular domain, a transmembrane region, and an intracellular kinase domain [17,18]. Numerous studies have shown that BAK1 is a multi-functional adaptor protein, interacting with diverse receptor-like kinase proteins involved in a variety of signaling pathways [19,20]. For example, in the case of brassinosteroid (BR)-mediated signaling, upon perception of BRs by the brassinosteroid-insensitive 1 (BRI1) receptor, which are important plant hormones for regulating multiple aspects of plant growth and development, BRI1 is activated in a phosphorylation-dependent manner and forms a heterocomplex with BAK1, in which BRI1 and BAK1 transphosphoralate each other sequentially, leading to the amplification of BR signaling pathway [21,22]. In Arabidopsis, overexpression of BAK1 results in enhanced BR signaling, accompanied by promoted plant growth, including narrower rosette leaves, longer petioles, and larger architecture, which resembles the phenotype of BRI-overexpression plants [17,18,21].

BAK1 has also been shown to be involved in some light-mediated physiological processes, in which BAK1 and its homologs (termed somatic embryogenesis receptor kinase (SERKs); BAK is also called SERK3) usually possess a functional redundancy [23,24,25]. Previous studies have shown that BAK1 and its closest homolog BKK1 double null mutant *bak1 bkk1* displays a BR-independent but light-dependent cell-death phenotype, suggesting BAK1 plays distinct roles in BR signaling and light-dependent cell-death signaling. Significantly, like many cell-death mutants, there is a significant accumulation of H_2_O_2_ and callose deposition in *bak1 bkk1*, accompanied by cell death [26,27]. In this study, we investigated the role of BAK1 in the high light-mediated plant growth. We found that overexpression BAK1 can suppress plant growth promoted by high light, presumably via attenuation of the cellular H_2_O_2_ level by phosphorylation of the scavenging catalases.

## 2. Results

### 2.1. Overexpression of BAK1 Suppresses the High Light-Promoted Plant Growth

**Figure 1:**
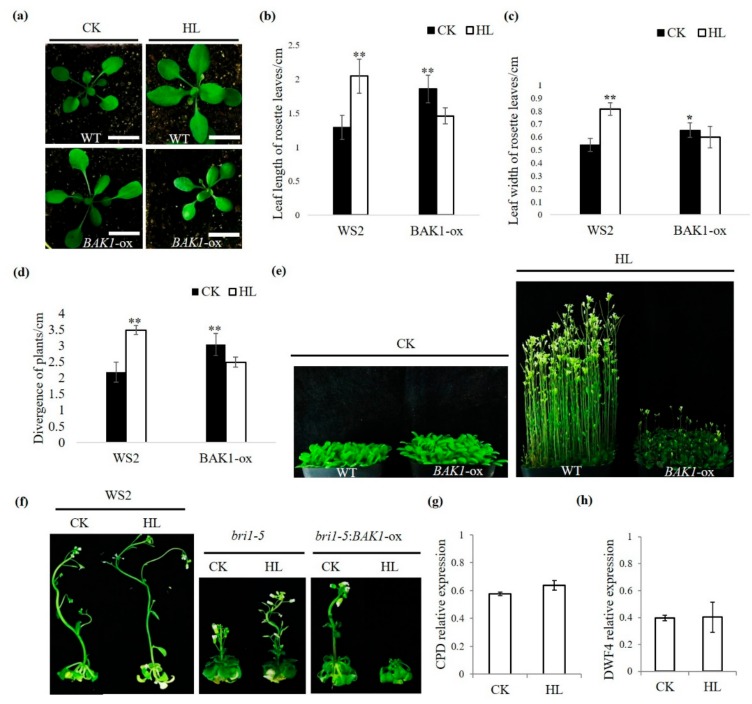
Overexpression of brassinosteroid-insensitive 1 (BRI1) associated receptor kinase 1 (BAK1) suppresses high light-promoted plant growth independent on the brassinosteroid-insensitive (BR) signaling pathway. (**a**) Representative Arabidopsis plants grown under different growth conditions. Seedlings were grown in soil for 8 days under 100 μmol·m^−^^2^s^−^^1^ light intensity and then subjected to high light treatment (400 μmol·m^−^^2^s^−^^1^) for 10 days. CK, control light; HL, high light. Scale bars = 1 cm. The experiments were repeated three times with similar results. (**b**–**d**) Quantitative analyses of leaf length, leaf width, and divergence of plants were made on at least 10 representative plants. Data represent means and standard errors. The asterisks indicate significant differences compared to the wild type plants in CK condition (*p < 0.05, **p < 0.01 by the Student’s *t*-test). The experiments were repeated three times. (**e**) Seedlings were grown for 8 days under 100 μmol·m^−^^2^s^−^^1^ light intensity and then subjected to high light treatment (400 μmol·m^−^^2^s^−^^1^) for 15 days. The experiments were repeated three times with similar results. (**f**) Representative plants grown on 1/2 MS medium under different growth conditions. Seedlings were grown for 8 days on 1/2 MS medium and then subjected to high light treatment (400 μmol·m^−^^2^s^−^^1^). The experiments were repeated three times with similar results. (**g**,**h**) Relative expression of *CPD* and *DWF4* genes in *bri1-5*:*BAK1*-ox plants grown under different conditions analyzed by qRT-PCR. The *ACTIN* gene was used as the reference gene. Error bars represent SD (*n* = 3).

Plant growth involves intricate regulatory programs that respond to various external factors and internal factors. Previous studies have shown that BAK1, as a co-receptor of BR signaling, plays a positive role in the regulation of plant growth, as manifested by the loss-of-function mutant *bak1* expresses a semi-dwarf phenotype, whereas overexpression of BAK1 in transgenic Arabidopsis renders the opposite phenotype with enlarged rosette [17,18,26]. In addition, a previous publication has shown, when Arabidopsis plants were grown under 400 μmol·m^−^^2^s^−^^1^ light intensity, the acclimation induced by high light renders higher rates of photosynthesis than plants grown under 100 μmolm^−2^s^−1^ light intensity [4]. Consistent with previous publications, we also found 400 μmol·m^−^^2^s^−^^1^ high light can promote Arabidopsis plant growth in both the vegetative and reproductive stages [4,28], including increased leaf length (Figure 1a,b) and leaf width (Figure 1a,c), enhanced divergence of plant (Figure 1a,d), and accelerated bolting (Figure 1e). To further investigate the role of BAK1 in plant growth, the BAK1 overexpression line in WS2 background (named *BAK1*-ox hereafter) was examined under high light condition (400 μmol·m^−^^2^s^−^^1^, 16 h light/8 h dark) and control growth condition (100 μmol·m^−^^2^s^−^^1^, 16 h light/8 h dark), respectively. We found, as reported previously, under control growth condition, overexpression of BAK1 promoted plant growth, as the high light did on the non-transgenic wild type (WT) plants: Plants exhibited longer leaves and enlarged rosette (Figure 1a–d). However, to our surprise, when the *BAK1*-ox plants were grown under the high light condition, the growth was significantly suppressed (Figure 1a–d), especially the reproductive growth-triggered by high light was partially arrested in the *BAK1*-ox plants (Figure 1e). These results suggest that overexpression of BAK1 interferes with growth-related cellular signaling activated by high light. 

### 2.2. The Suppression of BAK1 Overexpression on High Light-Promoted Plant Growth is Independent of the BR Signaling

Given the fact that BAK1 plays a positive role in the BR signaling that also regulates plant growth, we asked whether the high light-promoted plant growth is mediated by the BR signaling and whether the negative effect of overexpression of BAK1 on high light-promoted plant growth is dependent on BR signaling. We first examined the effect of high light on the BR signaling defective mutant. The *bri1-5* mutant (WS2 background) is a weak allele of *BRI1* and has some significant phenotypic differences compared to the WT plants, such as a severe dwarf stature, round rosette leaves, and short petioles [29] (Figure 1f). We treated the *bri1-5* mutant plants with high light and found that, as shown in Figure 1f, the inflorescent growth of *bri1-5* mutant plants were still significantly promoted by high light. To further demonstrate the relationship between suppression of BAK1 overexpression on high light-promoted growth and the BR signaling, the *bri1-5* background transgenic Arabidopsis overexpressing BAK1 (*bri1-5*:*BAK1*-ox) were generated and examined under high light treatment. As shown in Figure 1f, as in the WT background, when BAK1 was overexpressed in the *bri1-5* background, the enhanced inflorescent growth by high light was significantly repressed, suggesting overexpression of *BAK1* interferes with high light-promoted growth independent on the BR signaling pathway. 

The *CPD* and *DWF4* are two marker genes of the BR signaling pathway [30]. To further verify the suppression on high light-promoted growth by BAK1 overexpression does not attribute to the BR signaling, we determined the expression of the BR signaling marker genes in *bri1-5*:*BAK1*-ox plants with or without high light treatment. We did not find a significant difference of either marker gene expression upon high light treatment, again suggesting that *BAK1* overexpression suppresses high light-promoted plant growth via a BR-independent manner (Figure 1g,h).

### 2.3. High Light Enhances the BAK1 Phosphorylation Level in Plant Cells

**Figure 2:**
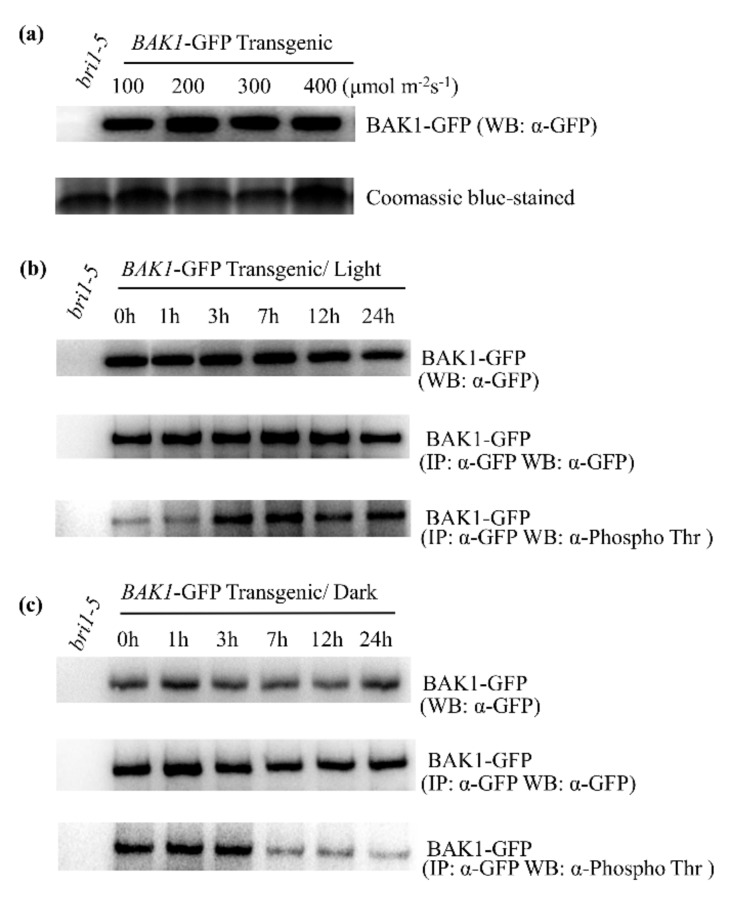
High light can enhance phosphorylation of BAK1 protein in vivo. (**a**) Western blotting analysis on *BAK1-GFP-*ox transgenic plants indicates similar accumulation levels of BAK1 protein when plants were grown under different light intensities. Coomassie blue staining indicates equal protein loading. The experiments were repeated three times. (**b**,**c**) The phosphorylation level of BAK1-GFP protein in *BAK1-GFP-*ox transgenic plants in response to high light treatment. (**b**) or darkness (**c**). The BAK1-GFP protein was immunoprecipitated from *BAK1-GFP-*ox transgenic plants or *bri1-5* control plants with anti-GFP antibody and protein A beads, followed by Western blotting analysis using anti-Phospho Thr antibody to detect the phosphorylation of BAK1-GFP. Phosphorylation of Thr residues in BAK1-GFP can be enhanced by high light treatment but attenuated dark treatment. The experiments were repeated three times.

The role of BAK1 in BR signaling has been well studied, in which the phosphorylation of BAK1 is essential for the activation of the BAK1 kinase [21]. Significantly, the BAK1 phosphorylation level is dramatically enhanced after plant perception of the BR elicitor [21]. To determine the role of BAK1 in plant response to high light, the phosphorylation level of BAK1 was examined upon high light treatment. To this end, we first determined whether light has any effect on the BAK1 protein stability. We extracted total protein from *bri1-5*:*BAK1*-*GFP*-ox plants treated with light with different intensities and analyzed BAK1 protein level by Western blotting analysis using the anti-GFP antibody. We found light does not have a significant impact on the BAK1 protein level (Figure 2a). We then determined possible phosphorylation alternation of BAK1 kinase in response to high light treatment. Ten-day-old *bri1-5*:*BAK1*-ox seedlings grown in 100 μmol·m^−^^2^s^−^^1^ light intensity were treated with 400 μmol·m^−^^2^s^−^^1^ high light and leaf tissues were collected at 0, 1, 3, 7, 12, and 24 h after treatment. The seedlings were then placed back in the dark, and leaf tissues were collected at 0, 1, 3, 7, 12, and 24 h afterward. The BAK1 protein was immunoprecipitation-purified by the anti-GFP antibody and protein A agarose beads from total protein extracted from leaf tissues. The immunoprecipitates were then separated on SDS-PAGE and detected with anti-GFP and anti-phosphothreonine antibody, respectively. Consistent with the result of the light intensity experiment, high light treatment had no significant effect on BAK1 protein accumulation (Figure 2b). However, upon strong light treatment, a gradually increased BAK1 phosphorylation signal was detected by the anti-phosphothreonine antibody within 3 h, suggesting high light stimulates the phosphorylation of BAK1 protein (Figure 2b). Consistent with this result of light-dependent phosphorylation of BAK1, when light treatment was terminated by placing plants in the dark, the phosphorylation of BAK1 was dramatically reduced within 7 h, even though the dark treatment did not alter the expression level of BAK1 protein (Figure 2c). Taken together, our results suggest that high light enhances the phosphorylation of BAK1.

### 2.4. Overexpression of BAK1 Enhances the Catalase Activity, Resulting in a Low Level of H_2_O_2_ in Plant Cells

**Figure 3:**
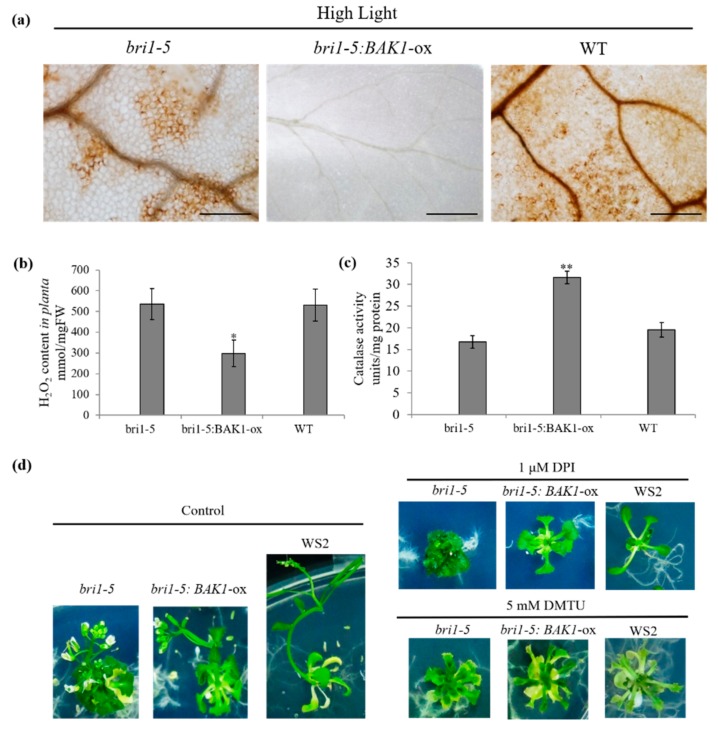
BAK1 overexpression plant under high light alleviates the H_2_O_2_ level through enhancing catalase activities. (**a**) DAB-staining of rosette leaves of WT, *bri1-5*, and *bri1-5:BAK1-ox* plants after high light treatment for 22 days. Bar = 200 μm. The experiments were repeated three times with similar results. (**b**,**c**) Catalase activity and H_2_O_2_ content in *bri1-5*, and *bri1-5:BAK1-*ox plants’ leaves after high light treatment for 22 days. Error bars represent SD (*n* = 4). The asterisks indicate significant differences compared to the wild type (WT) plants (*p < 0.05, **p < 0.01 by the Student’s *t*-test). The experiments were repeated three times. (**d**) Representative plants treated with diphenylene iodonium (DPI) or dimethylthiourea (DMTU) grown under 100 μmol·m^−^^2^s^−^^1^ light intensity. The 1 μM DPI and 5 mM DMTU were added to the 1/2 MS medium and seedlings were photographed after 22-day’s growth. The experiments were repeated three times with similar results.

Reactive oxygen species (ROS) have important roles in plant growth and development [9]. According to a previous publication [26], the *bak1bkk1* double mutant exhibited an early senescence phenotype under light, accompanied by H_2_O_2_ accumulation and spontaneous cell death. To explore the molecular mechanism of BAK1 overexpression-suppressed on the high light-promoted plant growth, a histochemical staining experiment was conducted to analyze the H_2_O_2_ concentration of plants in different growth conditions. As shown in Figure 3a, DAB staining indicated that, upon high light treatment, there was significant accumulation of H_2_O_2_ in the leaf of WS2 and *bri1-5* plants, but not in that of the *bri1-5*:*BAK1*-ox plant, which was further verified by quantification of H_2_O_2_ in leaf tissues (Figure 3b), suggesting that high light can trigger H_2_O_2_ burst in plants and such H_2_O_2_ burst may be attenuated by overexpression of BAK1. Since the cellular H_2_O_2_ level is normally tightly regulated by the scavenging enzyme catalase (CAT), we next determined the catalase activity in plants treated with high light. As shown in Figure 3c, the catalase activity in *bri1-5*:*BAK1*-ox plant leaves was much higher than that in *bri1-5* or WS2 plant leaves. Thus, these results suggest that overexpression of BAK1 attenuates the H_2_O_2_ burst triggered by high light, presumably via enhancement of the catalase activity, thereby eventually resulting in the arrest of plant growth.

Since overexpression of BAK1 significantly reduced H_2_O_2_ content in plant leaves, to further verify whether the reduced level of H_2_O_2_ is responsible for BAK1-mediated suppression on strong light-promoted plant growth, we artificially reduced the H_2_O_2_ level by blocking the generation of H_2_O_2_ by the addition of diphenylene iodonium (DPI), the inhibitor of NADPH oxidase responsible for the generation of H_2_O_2_; or by the removal of H_2_O_2_ using dimethylthiourea (DMTU) that can scavenge H_2_O_2_. The results showed that, with the application of DPI or DMTU, the growth of the WT *bri1-5*:*BAK1*-ox and *bri1-5* plants was dramatically suppressed (Figure 3d).

### 2.5. BAK1 Interacts with Three Catalases

**Figure 4:**
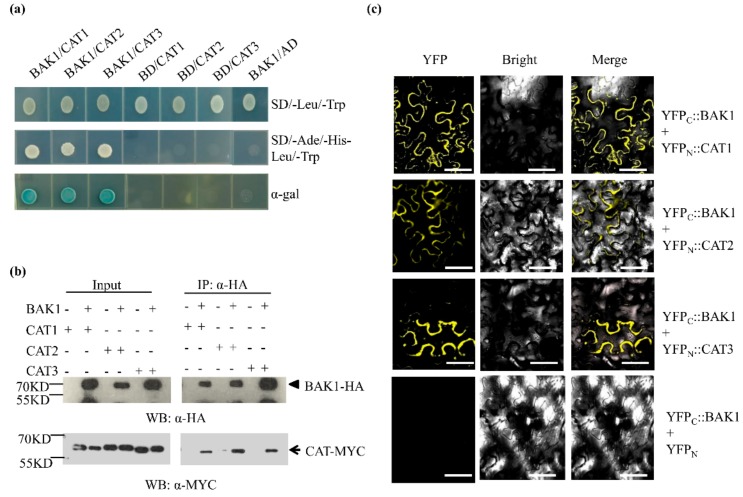
BAK1 interacts with three catalases. (**a**) Yeast two-hybrid assay of BAK1 interaction with Catalase 1 (CAT1), CAT2, and CAT3. The kinase domain of BAK1 was used as bait. BAK1/pGADT7 and pGBKT7/CATs were used as negative control. Yeast transformants were grown on the SD/-Ade/-His/-Leu/-Trp selection medium or X-galactosidase (X-Gal)-containing medium for 4 days before being photographed. The experiments were repeated three times with similar results. (**b**) In vivo interaction of BAK1 with catalase determined by co-immunoprecipitation (co-IP). *Agrobacterium tumefaciens* GV2260 strains containing the cauliflower mosaic virus 35S promoter-driven epitope-tagged BAK1 construct (BAK1-HA), CATs (CAT1-MYC, CAT2-MYC, and CAT3-MYC) were syringe-infiltrated into *N. benthamiana* leaves at a concentration of OD_600_ = 0.5. MG132 (25 μM) was conjected with the *Agrobacterium* suspension to enhance the amount of BAK1-HA. Proteins were extracted for immunoprecipitation with anti-HA affinity matrix one day after agrobacterial infiltration, followed by Western blotting using the anti-MYC antibody to determine the association of BAK1 with CATs. The presence of CATs protein (indicated by an arrow) in the immunoprecipitated complex of CAT expressed with BAK1 (indicated by an arrowhead) suggests specific interaction between them. The experiments were repeated three times with similar results. (**c**) BAK1 interacts with CATs in the cell membrane. The *A. tumefaciens* GV3101 strains containing the CaMV 35S promoter-driven BAK1-CYFP and CATs-NYFP constructs were co-expressed in *N. benthamiana* leaves. Three days after agrobacterial infiltration, epidermal cell layers were examined using a confocal microscope to capture the yellow fluorescent protein (YFP) signal resulting from the interaction between BAK1 and catalases. The experiments were repeated three times with similar results. Bar = 50 μm

We sought to explore the molecular factors associated with BAK1 by the affinity purification of the BAK1-containing protein complex in plant cells, followed by the LC-MS/MS analysis. To this end, total soluble proteins prepared from the 2-week-old seedlings of BAK1-GFP transgenic plants were incubated with the anti-GFP antibody and protein-A beads, and proteins eluted from the beads were separated by SDS-PAGE, followed by LC-MS/MS analysis to determine the identity of the BAK1-associated proteins. We found At1g20620 (CAT3) protein presenting in the BAK1 immunoprecipitated complex, suggesting that CAT3 is a candidate of BAK1-interacting protein in vivo. Since the Arabidopsis genome contains three catalase genes, namely *CAT1* (At1g20630), *CAT2* (At4g35090), and *CAT3* (At1g20620) [31], to verify the interaction of BAK1 with catalases, three catalase isoforms were used as prey for yeast two-hybrid assays. As shown in Figure 4a, when the BAK1 bait was co-transformed with the CAT1, CAT2, and CAT3 preys individually, the transformed yeast cells grew well on the selection medium (SD/-Ade/-His/-Leu/-Trp) and exhibited the X-galactosidase (X-gal) activity, indicating BAK1 interacts with CAT1, CAT2, and CAT3. 

We next verified the interaction of BAK1 with CAT1, CAT2, and CAT3 in plant cells by using a co-immunoprecipitation (co-IP) assay. The HA epitope-tagged BAK1 (35S::BAK1-HA) was co-expressed with the MYC epitope-tagged CAT1/2/3 (35S::CAT1/2/3-MYC) protein in *Nicotiana benthamiana* leaves via *Agrobacterium*-mediated transient expression. Two days after agroinfiltration, proteins were extracted for co-IP analysis. As shown in Figure 4b, CAT1-MYC, CAT2-MYC, and CAT3-MYC were detected in the anti-HA antibody-immunoprecipitated complex from the leaf tissues expressing CAT1/2/3-MYC and BAK1-HA, suggesting BAK1 interacts with CAT1/2/3 *in planta*. This result was further supported by our bimolecular fluorescence complementation (BiFC) assay, in which not only the subcellular localization of BAK1 and CAT1/2/3 but also the interaction of BAK1 with CAT1/2/3 were determined. Significantly, the BiFC assays showed that BAK1 can interact with all three catalases and the BAK1/CAT complex was localized to the plasma membrane (Figure 4c).

### 2.6. BAK1 Phosphorylates Three Catalases

**Figure 5:**
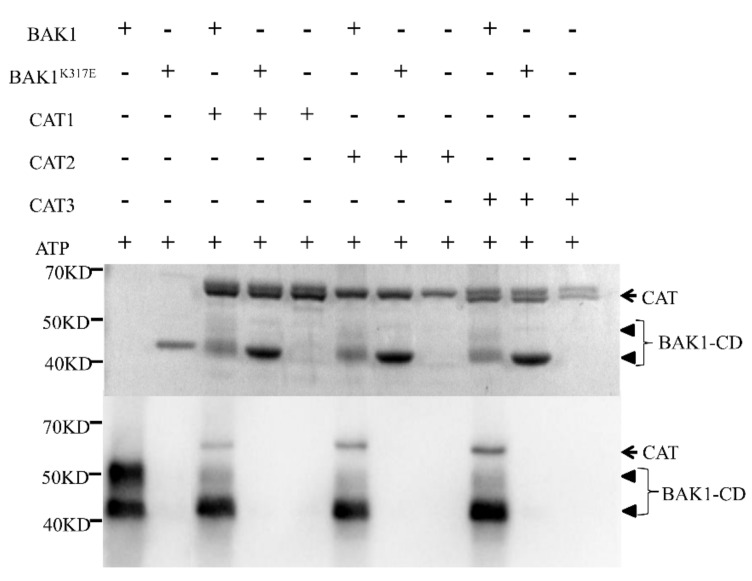
BAK1 can phosphorylate CATs in vitro. The Flag-BAK1-CD (CD, cytoplasmic domain), Flag-BAK1^K317E^-CD, and Flag-CAT1/2/3 constructs were expressed in *E. coli* BL21 and the recombinant proteins were purified using anti-Flag affinity gel. In vitro phosphorylation reaction was carried out using appropriate recombinant proteins and separated by SDS-PAGE. Coomassie blue-stained recombinant BAK1-CD, BAK1^K317E^-CD, and CAT1/2/3 proteins are shown in the top panel. The phosphorylation assay is shown in the bottom panel. The arrowhead shows BAK1 and the arrow shows CAT1/2/3 proteins. The phosphorylation of CAT1/2/3 could be detected after it reacted with BAK1-CD. The experiments were repeated three times with similar results.

The kinase feature of BAK1 and its interaction with CAT1/2/3 gave rise to a hypothesis that CAT1/2/3 could be the substrate of BAK1 kinase. To test this notion, we conducted the in vitro kinase assay to determine whether BAK1 can phosphorylate CAT1/2/3. The recombinant CAT1/2/3, BAK1, and BAK1^K317E^, a kinase-deficient mutant served as the negative control, were expressed in and purified from *Escherichia coli* for the phosphorylation reaction. The results showed that BAK1 not only possesses autophosphorylation activity but also phosphorylates all three catalases in vitro (Figure 5), indicating CAT1/2/3 is a substrate of the BAK1 kinase.

Furthermore, we sought to determine the amino acid residues in CAT1/2/3 that are phosphorylated by BAK1. To this end, the in vitro phosphorylation reaction mixture was separated by SDS-PAGE, and the Flag-CAT1/2/3 protein bands were excised and subjected to Orbitrap LC-MS/MS. Multiple BAK1-mediated phosphorylation sites were identified by MS assay in different motifs of CAT1/2/3 (Figure S1), which was predicted by the MEME Suite [32]. As shown in Table 1, we identified eight amino acid residues of CAT1 phosphorylated in vitro by BAK1, including Y45 in motif A, T78/T85/S86 in motif C, S351 in motif L, T409 in motif M, and T460/S477 in motif O, respectively; CAT2 had five amino acid residues phosphorylated by BAK1, including S30 in motif A, S205 in motif G, T352 in motif L, T408 in motif M, and T456 in motif O, respectively; CAT3 had seven amino acid residues phosphorylated by BAK1, including S31 in motif A, S82 in motif C, T128 in motif E, T273 in motif I, and S395/T408/S412 in motif M, respectively. Interestingly, we found conserved amino acid residue in CAT1/2/3 phosphorylated by BAK1, suggesting a distinct phosphorylation pattern of CAT1/2/3 by the BAK1 kinase.

### 2.7. BAK1 Overexpression Effect on Suppressing High Light-Promoted Growth is Abolished by Knockout of Catalase Genes

**Figure 6:**
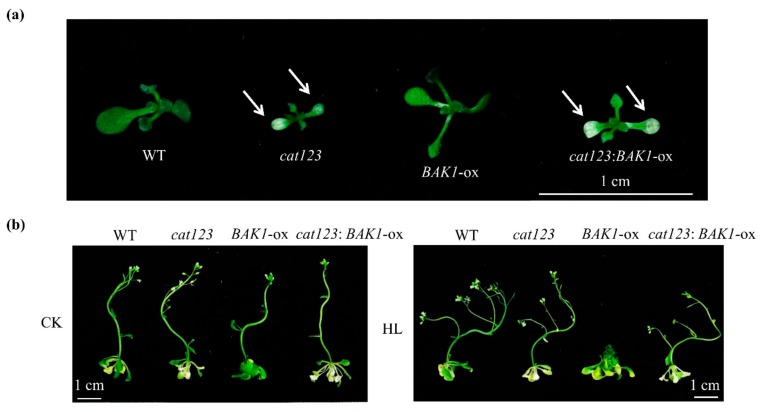
Knockout of catalase genes abolishes BAK1 overexpression-mediated suppression on plant growth-promoted by high light. (**a**) Seedlings of the *cat123* and *cat123:BAK1-ox* triple mutants generated by CRISPR-Cas9. Arabidopsis seedlings with different genetic backgrounds were grown for 10 days before being photographed. The arrow shows leaf bleaching. The experiments were repeated three times with similar results. (**b**) Knockout of catalase genes in *BAK1-ox* plants restored growth promoted by high light. Seedlings were grown for 8 days on 1/2 MS medium and then treated with high light (400 μmol·m^−^^2^s^−^^1^) for 22 days before being photographed. Scale bars = 1 cm. The experiments were repeated three times with similar results.

Given the fact that BAK1 phosphorylates CAT1/2/3, it is possible that CAT1/2/3 play significant roles in the BAK1-dependent suppression on strong light-promoted plant growth. To test this hypothesis, we sought to genetically knockout *CAT1/2/3* genes in the WT and *BAK1*-ox background by using the CRISPR/Cas9 gene-editing technique. The gRNA specifically targeting all three *CAT* genes was determined and cloned into *pHSN401*, followed by Agrobacterium-mediated transformation to generate *cat123* triple mutants (Figure S2a). The gene-editing results of each *CAT* genes in different backgrounds are shown in Figure S2b. We found that similar to the phenotype of *cat2* single mutant and *cat1 cat2*/*cat2 cat3* double mutant reported previously [33,34,35], the *cat1 cat2 cat3* triple mutants showed leaf bleaching that is a typical feature of catalase-deficient mutant (Figure 6a). Significantly, the catalase activity was decreased in the *cat123* triple mutant compared to that in corresponding background plants (Figure S3a), and, in contrast, the H_2_O_2_ level *in planta* was increased in *cat123* triple mutant (Figure S3b). We determined the reproductive growth of *cat123* triple mutants in the high light condition and control light condition. As shown in Figure 6b, under control light condition, the reproductive growth of all tested plants did not show a significant difference. However, upon high light treatment, unlike *BAK1*-ox, the *cat123*:*BAK1*-ox plant grew indistinguishably from WT or *cat123* mutant plants, showing recovery of high light-promoted reproductive growth (Figure 6b). This suggests the knockout of the *Catalase* genes completely abolishes the BAK1’s impact on high light-promoted growth. Thus, we conclude that BAK1 regulates high light-enhanced growth in a catalase-dependent manner.

## 3. Discussion

BAK1 is considered as an adaptor-like receptor kinase that can form a receptor complex with multiple membrane ligand-binding receptor kinases to participate in a variety of signaling pathways [20]. In the case of regulating plant growth, many publications have shown that overexpression of BAK1 in transgenic plants significantly promotes growth, in which it is generally thought that such phenomenon is due to the enhanced BR signaling via BAK1 overexpression [17,18,21,36,37]. However, it has also been reported that extreme overexpression of BAK1 by the 2×CaMV 35S promoter in *Arabidopsis thaliana* causes detrimental effects on various aspects of plant development, including growth arrest, leaf necrosis, and reduced seed production [38]. Here, we reported for the first time that overexpression of BAK1 suppresses the growth promoted by high light in Arabidopsis (Figure 1), revealing a novel role of BAK1 in high light-mediated regulation of plant growth and development.

Light is a very important factor in regulating plant growth and development, and both light intensity and light spectrum are important components of the light signal [1,2]. In our study, we found that 420–720 mm light could enhance the BAK1 phosphorylation signal. There are a lot of photoreceptors in plants, playing essential roles in precepting the light signal and transferring the signal into cells [39]. Based on Zoratti et al. [40], cryptochromes, phototropins, and phytochromes play important roles in percept 420–720 mm light. We suspect that BAK1 may involve in these photoreceptors’ mediating signaling pathways. However, our primary experiment, which applied red and blue light, showed that the light quality did not make a significant difference to the specific phenotype.

It has also been found previously that BAK1 plays a role in the regulation of H_2_O_2_ levels in plant cells. In the BAK1-mediated BR signaling, BR functions through the BRI1/BAK1 complex to modulate the activity of NADPH oxidase for the production of H_2_O_2_, thereby regulating the process of plant growth and stress response [41,42,43]. In addition, the *bak1bkk1* double mutant exhibited an early senescence phenotype under light, accompanied by H_2_O_2_ accumulation; in fact, *bak1bkk1* displayed spontaneous cell death even under sterile growing conditions [26]. Interestingly, no significant alternation to the H_2_O_2_ level was found in the *bak1* single mutant, suggesting BAK1 and BKK1 function together to regulate H_2_O_2_ accumulation [26,27]. Here we provide evidence showing that BAK1 controls H_2_O_2_ levels in plant cells via a BR-independent signaling pathway through interacting with and phosphorylating catalase enzymes.

H_2_O_2_, as an important form of ROS, plays an important role in many physiological processes, including defense response, abiotic stresses, senescence, and growth and development. A growing body of evidence has shown that homeostasis of H_2_O_2_ is critical for maintaining proper physiological processes, particularly H_2_O_2_ levels have a significant impact on plant growth and development. In fact, we have tested the in vitro supplementation of H_2_O_2_ effect on plant growth and found that 2.5 mM H_2_O_2_ could significantly promote plant rosette part growth, especially the leaf length and the divergence of plant, but 7.5 mM H_2_O_2_ showed toxic activity significantly inhibiting plant growth (Figure S4). These results indicated certain levels of H_2_O_2_ can promote plant growth and development, and the effect of H_2_O_2_ on plant growth and development is complex. In addition, the H_2_O_2_ level significantly increases during bolting and flowering in Arabidopsis, which is mainly attributed to a decline of CAT2 activity [11,44]. There are three hydrogen peroxide scavenging enzymes in Arabidopsis, CAT1, CAT2, and CAT3, that regulate the cellular level of H_2_O_2_. In our study, we found, despite the underlying mechanism still being largely unknown, high light enhances the activity of three CATs, resulting in less accumulation of H_2_O_2_ in plant cells, thereby leading to the arrest of reproductive growth. These results are consistent with the further observation that artificial reduction in H_2_O_2_ content *in planta* via exogenous application of NADPH oxidase or H_2_O_2_ scavenger suppresses plant growth as well (Figure 3d). Naturally, the high light enhanced BAK1 phosphorylation and then activated CATs activity to degrade the extra production of H_2_O_2_ to maintain a proper balance. However, BAK1 overexpression enhanced CAT activity in bias and disturbed the homeostasis of H_2_O_2_ to cause the suppression of plant growth and development.

To investigate the relationship between catalase and BAK1, we found that BAK1 interacted with all three CATs and, more importantly, phosphorylates them in vitro. Phosphorylation has been demonstrated as an important posttranslational modification in many RLKs, including BAK1, to regulate diverse cellular signaling pathways [45]. We found the phosphorylation of BAK1 was significantly enhanced when Arabidopsis was treated with high light. Given the fact that the phosphorylation of BAK1 is generally necessary for its potential activation of signaling, such as BR signaling [21,22] and innate immune signaling [46,47], we speculate that high light enhances phosphorylation of BAK1 for its activity potential, which in turn phosphorylates CAT1/2/3. It is notable that in Arabidopsis after CAT3 is phosphorylated by a calcium-dependent protein kinase, the catalytic activity of CAT3 was significantly enhanced [48], and in rice, the RLCK protein STRK1 phosphorylates CatC to stimulate its activity to regulate H_2_O_2_ homeostasis and improve salt tolerance [49]. Thus, our results give rise to a hypothesis that phosphorylation of CATs by BAK1 results in the activation of CATs to scavenge H_2_O_2_, consequently leading to a reduction in H_2_O_2_ levels. This hypothesis is supported by further genetic analysis. When all three *CAT* genes were knocked out by CRISPR-Cas9-mediated gene editing, the suppression of BAK1 overexpression-conferred suppression of high light-promoted growth was completely abolished (Figure 6b), suggesting that the *CAT* genes are the essential downstream components for BAK1 to suppress high light-promoted plant growth.

**Figure 7:**
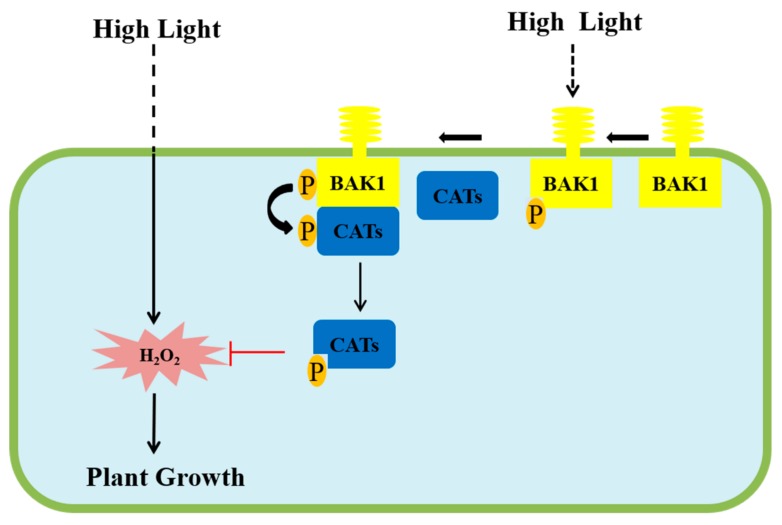
A proposed simplified model for the overexpression of BAK1 inhibiting high light-induced plant growth and development. High light can induce certain levels of H_2_O_2_ accumulation which will eventually lead to the promotion of growth and development of Arabidopsis plants. In the transgenic Arabidopsis overexpressing *BAK1*, high light can also induce the phosphorylation of the over-accumulated BAK1 kinase caused by the expression of the *BAK1* gene, which in turn interacts with and phosphorylates catalases CAT1/2/3, resulting in the activation of CAT1/2/3. The activated catalase acts in scavenging H_2_O_2_, thereby leading to the arrest of high light-promoted growth and development.

In summary, we propose a simplified model based on our research results (Figure 7): In the transgenic Arabidopsis overexpressing *BAK1*, high light can induce the phosphorylation of the over-accumulated BAK1 kinase caused by the expression of the *BAK1* gene, which in turn interacts with and phosphorylates catalases CAT1/2/3, resulting in activation of CAT1/2/3. The much-activated catalases act in scavenging H_2_O_2_, thereby leading to the arrest of high light-promoted growth and development. In the wildtype Arabidopsis with BAK1 natural expression, high light inducing H_2_O_2_ accumulation properly is controlled by light-activated BAK1 to maintain the H_2_O_2_ homeostasis, which will eventually lead to the promotion of growth and development of Arabidopsis plants.

## 4. Materials and Methods

### 4.1. Plant Materials and Growth Conditions

Wild-type Arabidopsis plants used in this study were Wassilewskija-2 (WS2) ecotype. The Arabidopsis transgenic lines *bri1-5*:*BAK1*-ox and *BAK1*-ox (BAK1 protein were fused at the C-terminal of GFP tag with the constitutive cauliflower mosaic virus (CaMV) 35S promoter) were obtained from Jia Li group [26,27]. Other Arabidopsis lines were conserved or established in our Laboratory.

For seedlings planting in the potting soil mixture, seeds were vernalized for 2 days at 4 °C and transferred to a growth chamber. For plants planted in dishes, seeds were sterilized in 15% chlorine bleach (10% available chlorine) for 15 min. After sterilization, seeds were rinsed 3–5 times with sterile water and germinated on plates containing one-half MS medium (Nissui, Qingdao, China), 2% sucrose, and 0.7% agar. Plants were grown at 22 °C under 16 h light/8 h dark unless otherwise specified.

### 4.2. Light Treatment Conditions

The seedlings were precultured at 100 μmol·m^−^^2^s^−^^1^ light intensity for 8 days. After that, the seedlings were treated with 400 μmol·m^−^^2^s^−^^1^ light intensity for high light treatment, while the control group was grown under 100 μmol·m^−^^2^s^−^^1^ light intensity. The light incubator we used for light treatment was GDN-800D-4 which is produced by the NINGBO SOUTHEAST INSTRUMENT CO (Ningbo, China)., LTD. The light source in this incubator was white light emitting diode (LED) lamps, which spectrum is 420–720 mm.

### 4.3. Statistical Analysis

Microsoft Excel and PASW Statistics (version 18) were used for statistical analyses. Data were subjected to standard deviation analysis, and a comparison was carried out by Student’s *t*-test (*p < 0.05, **p < 0.01).

### 4.4. Protein Phosphorylation Level Assay In Vivo

Arabidopsis seedlings were grown in a shaking liquid culture, as previously described [21]. Samples were collected after 10 days’ cultivation, then ground very fine with liquid nitrogen. Total protein was extracted in 2× extraction buffer, 100 mM Tris-HCl, pH 7.4, 300 mM NaCl, 10 mM EDTA, 20% glycerol, 0.4% Triton X-100, 40 mM NaF, 2 mM PMSF, and protein inhibitor (Roche, Mannheim, Germany). Extracts were centrifuged at 13,000 g at 4 °C for 15min to collect the soluble fraction. The supernatant was taken, and the concentration measured and 10 mg protein was incubated with anti-GFP (Life Technology, Carlsbad, CA, USA) for 3 h using a rotator. After that, protein A agarose beads (Transgen Biotech, Beijing, China) were added into the reaction mixture and agitated overnight. Then the beads were precipitated with 1000 g at 4 °C. The beads were washed 3 times with 1× extraction buffer. Bound proteins were eluted by boiling the beads in 1.5× SDS protein loading buffer and the purified proteins were identified by Western blot.

### 4.5. Western Blot Analysis

Proteins were separated with 10% sodium dodecyl sulfate (SDS) polyacrylamide gel electrophoresis. BAK1-GFP and BAK1 proteins’ phosphorylation level was detected respectively by GFP Antibody (Transgen Biotech, Beijing, China) and P-Threonine Antibody (Cell Signaling Technology, Danvers, MA, USA) as previously described [21,22]. BAK1-HA and CAT-MYC were detected, respectively, by HA Antibody (Sigma, Saint Louis, MO, USA) and MYC Antibody (Sigma, Saint Louis MO, USA).

### 4.6. Mass Spectrometry

IP proteins were fractionated on SDS-PAGE gels and stained in Coomassie Blue dyeing liquor. The target bands were cut and then broken into 3-6 bands. Using decolorizing solution buffer (50 mM NH_4_HCO_3_, 50% acetonitrile), the target bands were shaken and decolorize to colorless. Acetonitrile was added to dehydrate the gel bands to white. Water was added for gel bands’ recovery and washing. Fifty micromolar NH_4_HCO_3_ was added to provide conditions for trypsin activity. Three hundred nanograms of trypsin was added into the sample (diluted with 50 mM NH_4_HCO_3_). The sample was vacuum dried. The sample was reconstituted with 0.1% formic acid. Samples were analyzed on Orbitrap Fusion, and a Q Exactive HF mass spectrometer (Thermo Fisher Scientific, Rockford, IL, USA) connected to an UltiMate 3000 RSLCnano System (Thermo Fisher Scientific, Rockford, IL, USA). Dried peptide samples were re-dissolved in Solvent A (0.1% formic acid in water) and loaded to a pre-column (100 μm × 2 cm, home-made; particle size, 3 μm; pore size, 120 Å; SunChrom, USA), then separated on a home-made 150 μm × 15 cm silica microcolumn (particle size, 1.9 μm; pore size, 120 Å; SunChrom, USA) with a gradient of 6–40% mobile phase B (0.2% formic acid in acetonitrile) at a flow rate of 600 nl/min for 75 min at room temperature. A data-dependent strategy was used by measuring MS1 in the Orbitrap at a resolution of 120,000, followed by tandem MS scans of the top 20 precursors using higher-energy collision (HCD) dissociation with 27% of normalized collision energy and 18 s of dynamic exclusion time.

### 4.7. Physiological Biochemical Index Measurement in Planta

The hydrogen peroxide content and catalase activity measurement *in planta* were used with a Hydrogen peroxide assay kit, Catalase (CAT) assay kit (Visible light) (Nanjing Jiancheng Bioengineering Institute, Nanjing, China), respectively.

Tissue staining with DAB (1 mg/mL, Sigma, Saint Louis, MO, USA) was carried out as reported previously [26,27].

### 4.8. RNA Extraction and qRT-PCR

Total RNA was extracted from 14-day-old seedlings using the RNAiso Plus Kit (TaKaRa, Mountain, CA, USA), and the Transcriptor First Strand cDNA Synthesis Kit (Roche, Mannheim, Germany) was used for reverse transcription. Quantitative real-time PCR (qRT-PCR) was performed with an SYBR Green PCR Master Mix kit (Vazyme, Nanjing, China). Relative expression levels were measured and calculated with Step One Plus™ (Applied Biosystems, Beverly, MA, USA), and the specific primers used are listed in Supporting Information Table S1.

### 4.9. Yeast Two-Hybrid Assays

To make the yeast two-hybrid assays constructs, the full-length cDNA fragments of CAT1, CAT2, and CAT3 were PCR amplified, respectively, and cloned into vectors pGADT7 via EcoR I, BamH II. The sequence encoding the BAK1 kinase domain was cloned into vector pGBKT7. Obtained constructs were sequenced to ensure there was no nucleotide change or substitution during PCR amplification. Desired pairs of constructs were co-transformed into AH109 yeast cells to examine the interactions of relevant proteins. The positive clones were identified by the criteria that yeast cells can grow on SD/-Ade/-His/-Leu/-Trp medium and have X-galactosidase activities. The primer sequences and restriction sites are listed in Supporting Information Table S1.

### 4.10. Co-IP Assay

*Agrobacterium tumefaciens* GV2260 strains containing the cauliflower mosaic virus 35S promoter-driven epitope-tagged BAK1 construct (BAK1-HA), CATs (CAT1-3-MYC) construct were syringe-infiltrated into *Nicotiana benthamiana* leaves at a concentration of OD_600_ = 0.5. MG132 (25 μM) was co-injected with the *Agrobacterium* suspension to prevent the degradation of BAK1-HA protein. One day after *Agrobacterium* infiltration, proteins were extracted for immunoprecipitation (IP) with α-HA affinity matrix, as described before [50], followed by Western blotting (WB) using the α-MYC antibody.

### 4.11. Subcellular Localizations

Subcellular localization assays were performed as described previously [51]. For the generation of GFP-fusion protein, the coding sequence of each CAT was constructed into a vector Cam-35S-GFP vector. All constructs were transformed into the *Agrobacterium strain* GV3101. The *Agrobacterium* lines were inoculated into the abaxial sides of the leaves of 3-week-old *N. benthamiana*. Samples were collected 3 to 4 days after inoculation.

### 4.12. BiFC assays

BiFC assays were performed as described previously [52]. For the generation of the BiFC vectors, the coding region of BAK1 was cloned via *Kpn* I and *Bam*H I into pSPYCE (M), resulting in BAK1-CYFP, and the coding region of CAT1, CAT2, or CAT3 was cloned via *Kpn* I and *Bam*H I into pSPYNE-173, resulting in CAT1-NYFP, CAT2-NYFP, and CAT3-NYFP, respectively. All constructs were transformed into the *Agrobacterium* strain GV3101. An equal volume of *Agrobacterium* harboring BAK1-YC, CAT1-YN (or CAT2-YN, CAT3-YN), and P19 was mixed to a final concentration of OD_600_ = 0.8. Agrobacterium lines were infiltrated into leaves of *N. benthamiana*. Plants were grown at 22 °C and allowed to recover for 3 days; then, the fluorescence of YFP in the leaves was imaged using a confocal laser scanning microscope (Olympus FV1000).

### 4.13. In Vitro Kinase Assay

Protein purification in vitro was used with *Escherichia coli* Amino-terminal FLAG^®^ Expression Kit (Sigma, Saint Louis, MO, USA). Generation of Flag-BAK1-CD (CD, cytoplasmic domain) and Flag-BAK1^K317E^-CD is described as previously [21]. The coding sequence of each CAT was amplified from the right construct pGADT7:CAT, and cloned via *Hin*d III and *Kpn* I sites into the pFLAG-MAC vector. The constructed vectors were transformed into BL21 and induced by IPTG for protein expression. After harvesting cells, the cells were sonicated for breaking. The supernatant solution was collected by centrifugation. Anti-flag M2 affinity gel (Sigma, Saint Louis, MO, USA) was added to the supernatant to purify the protein. Elution buffer (50 mM Tris-HCl, pH 7.5, 150 mM NaCl, 0.3 mg/mL Flag peptides, 0.1% Triton X-100) was used for obtaining the target protein. The corresponding target protein was reacted in the kinase buffer (50 mM HEPES-KOH, PH 7.4, 10 mM MgCl_2_, 10 mM MnCl_2_, 1 mM DTT and 0.2 mM unlabeled ATP) for 1 h. Reactions were terminated by adding the same volume 2× SDS loading buffer. The primer sequences and restriction sites are listed in Table S1.

### 4.14. Generation of cat123 Triple Mutants

To generate *cat123* triple mutants in WT, and *BAK1*-ox backgrounds, a CAT1/2/3 conserved target, a CAT1/2 conserved target, or a CAT3 specific target were selected as double targets for Cas9 to mutate CAT1/2/3 at the same time. Vector construction and mutant identification were performed as described [53,54]. The targets were cloned into the pHSN401 vector via *Bas* I. The constructs were introduced into WT, and *BAK1*-ox backgrounds by floral dip, respectively. The resulting transgenic T1 seeds were screened on 1/2 MS medium containing 33 mg/mL hygromycin. Genomic fragments covering the mutation sites were amplified from the T1 transgenic plants by PCR and sequenced. T1 heterozygous mutants were kept for the T2 generation to identify homozygous mutants. The mutant seeds were harvested from individual lines to obtain T3 seeds. The seeds were screened with hygromycin, and nonhygromycin resistant seeds were used for the following experiments.

## Figures and Tables

**Table 1:** Identification of Catalase (CAT) Phosphorylation Sites by LC/MS/MS in vitro.

Peptide	Identified Site
BAK1-CD+CAT1	
GPILLEDyHLLEK	Y45
GFFEVtHDITQLtSADFLR	T78, T85
GFFEVTHDITQLTsADFLR	S86
IFSYADsQR	S351
HAEKYPTtPIVCSGNR	T409
FVEALSEPRVtHEIR	T460
SIWISYWSQADKsLGQK	S477
BAK1-CD+CAT2	
YRPASSYNSPFFTTNSGAPVWNNNsSMTVGPR	S30
HMDGsGVNTYMLINK	S205
VFSYADtQR	T352
DEEVNYFPsRYDQVR	S395
HAEKYPtPPAVCSGK	T408
BAK1-CD+CAT3	
YRPSSAYNAPFYTTNGGAPVSNNISsLTIGER	S31
GFFEVTHDIsNLTCADFLR	S82
GFFEVTHDIsNLTCADFLRAPGVQTPVIVR	S82
FYtREGNFDLVGNNTPVFFIR	T128
LFIQtMDPADEDKFDFDPLDVTK	T273
DEEINYYPsKFDPVR	S395
VPTPTNsYTGIR	S412

## Supplementary Materials

Supplementary materials can be found at https://www.mdpi.com/1422-0067/21/4/1437/s1.
