# Supplementary material for: BAK1 Mediates Light Intensity to Phosphorylate and Activate Catalases to Regulate Plant Growth and Development
Source: Int J Mol Sci. 2020 Feb 20;21(4):1437. doi: 10.3390/ijms21041437 (PMC7073115; doi:10.3390/ijms21041437)
Supplement: Supplementary file 1 [file ijms-21-01437-s001.pdf]

Supplementary Materials:

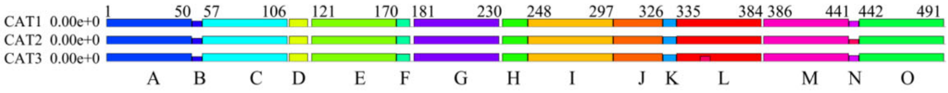

Figure S1. Functional motifs of three Arabidopsis CATs. The motif was predicted at http://meme-suite.org/. This diagram shows the location of motif sites. Each block shows the position and strength of a motif site. The height of a block gives an indication of the significance of the site as taller blocks are more significant. The height is calculated to be proportional to the negative logarithm of the *p*-value of the site, truncated at the height for a *p*-value of 1e^{-10}.

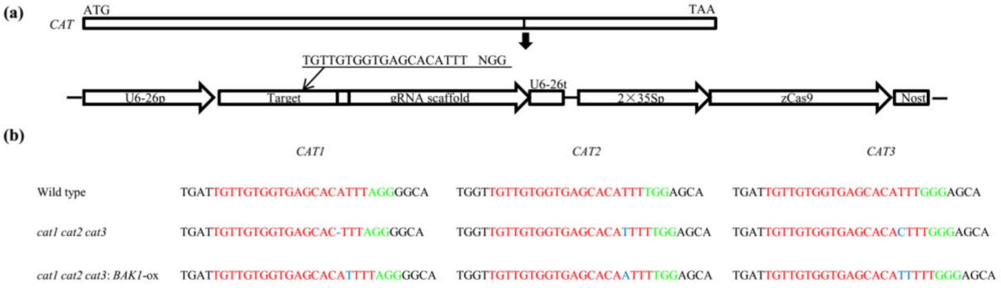

Figure S2. Generation of triple mutant *cat123* Arabidopsis plants. (a) Schematic of the *cat123* triple mutant. (b) Three *CAT* genes' editing results in WT and *BAK1*-ox backgrounds. The PAM sequence is highlighted by green color. The sgRNA is highlighted by red color. The mutation sites are highlighted by blue color.

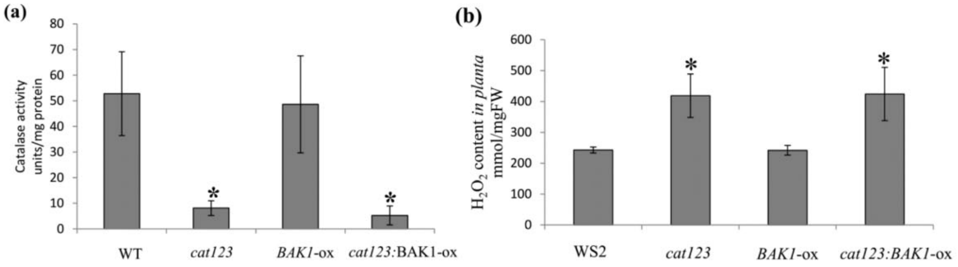

Figure S3. Catalase activity and H_2O_2 content *in planta*, which were measured in 10-day-old Arabidopsis seedlings. Error bars represent SE (n = 4). The asterisks indicate significant differences compared to the wild type (*P < 0.05 by the student's t-test). The experiments were repeated three times.

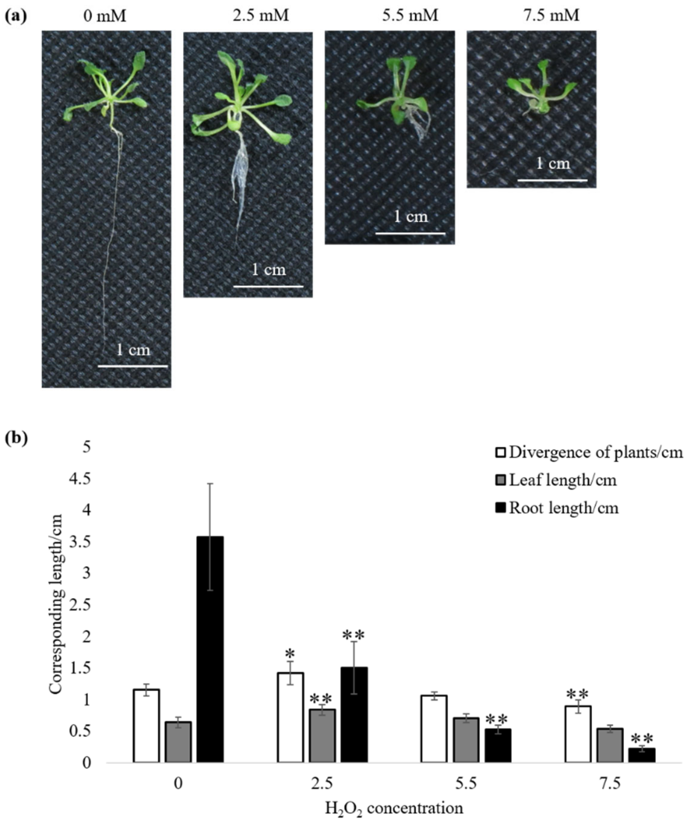

Figure S4. Effect of *in vitro* supplementation of H_2O_2 on plant growth. (a) Representative WT Arabidopsis plants treated with 0 mM, 2.5 mM, 5mM, 7.5 mM H_2O_2 concentration grown under 100 μmol $m^{-2}s^{-1}$ light intensity. The H_2O_2 were added in the 1/2 MS medium and seedlings were photographed after 17-day growth. The experiments were repeated three times with similar results. (b) Quantitative analyses of divergence of plants, leaf length, and root length, which were conducted using at least 10 representative plants. Data represent means and standard errors. The asterisks indicate significant differences compared to plants with 0 mM H_2O_2 treatment (**$P < 0.01$, *$P < 0.05$ by the student's t-test). The experiments were repeated three times.

Supplemental Table 1. Primer Sequences Used in This Study.

Purpose	Primer name	sequence (5'-3')
Cloning of CAT	CAT1-KpnⅠ-F	CGGGGTACCATGGATCCATACAGGGT
	CAT1-SalⅠ-R	ACGCGTCGACGAAGTTTGGCCTCACGTTAAGAC
	CAT2-3UTR-F	GGCCAATCAAGAATTCTTTCACT
	CAT2/3-KpnⅠ-F	CGGGGTACCATGGATCCTTACAAGTA
	CAT2-SalⅠ-R	ACGCGTCGACGATGCTTGGTCTCACGTTCAGA
	CAT3-SalⅠ-R	ACGCGTCGACGATGCTTGGCCTCACGTTC
RT-qPCR	DWF4 qRT-F	CCACAACACTCGGTGACTTCA
	DWF4 qRT-R	TCAGCTGATACGATCGTTGGTT
	CPD qRT-F	TGAGACGCTACGAGTGGCTAAC
	CPD qRT-R	GCATCTTTGAAGTGGTTTGGG
	Actin2-F	TGTGCCAATCTACGAGGGTTT
	Actin2-F	TTTCCCGCTCTGCTGTTGT

Yeast two hybrid assay	CAT1-ADEcoR Ⅰ -F:	GCCATGGAGGCCAGTGAATTCATGGATCCATACAGGGTTCGTC
	CAT1-ADEcoR Ⅰ -R:	CAGCTCGAGCTCGATGGATCCGAAGTTTGGCCTCACGTTAAGAC
	CAT2-ADEcoR Ⅰ -F:	GCCATGGAGGCCAGTGAATTCATGGATCCTTACAAGTATCGTCCA
	CAT2-ADEcoR Ⅰ -R:	CAGCTCGAGCTCGATGGATCCGATGCTTGGTCTCACGTTCAGA
	CAT3-ADEcoR Ⅰ -F:	GCCATGGAGGCCAGTGAATTCATGGATCCTTACAAGTATCGTC
	CAT3-ADEcoR Ⅰ -R:	CAGCTCGAGCTCGATGGATCCGATGCTTGGCCTCACGTTCAGAC
Protein expression	PflagMAC-HindIII-CAT1-F	ATGACAAAGTCAAGCTTATGGATCCATACAGGGTTCGTC
	PflagMAC-Kpn Ⅰ -CAT1-R	CTATCTAGATCTGCAGGTACCGAAGTTTGGCCTCACGTTAAGAC
	PflagMAC-HindIII-CAT2-F	ATGACAAAGTCAAGCTTATGGATCCTTACAAGTATCGTCCA
	PflagMAC-Kpn Ⅰ -CAT2-R	CTATCTAGATCTGCAGGTACCGATGCTTGGTCTCACGTTCAGA
	PflagMAC-HindIII-CAT3-F	ATGACAAAGTCAAGCTTATGGATCCTTACAAGTATCGTCCTT
	PflagMAC-Kpn Ⅰ -CAT3-R	CTATCTAGATCTGCAGGTACCGATGCTTGGCCTCACGTTC
Crispr/CAS9	T1-BS-AtCAT123-1-F	ATATATGGTCTCTATTGTGTTGTGGTGAGCACATTTGTT
	T1- AtCAT123-1-F	GTGTTGTGGTGAGCACATTTGTTTTAGAGCTAGAAATAGC
	T2-BS-AtCAT123-1-R	ATTATTGGTCTCTAAACACAAGATCAAAGTTCCCCTCAA
	T2- AtCAT123-1-R	CACAAGATCAAAGTTCCCCTCAATCTCTTAGTCGACTCTAC
	T1-BS-AtCAT123-2-F	ATATATGGTCTCTATTGTGTTGTGGTGAGCACATTTGTT
	T1- AtCAT123-2-F	GTGTTGTGGTGAGCACATTTGTTTTAGAGCTAGAAATAGC
	T2-BS-AtCAT123-2-R	ATTATTGGTCTCTAAACACAAGATCAAAGTTTCCCTCAA
	T2- AtCAT123-2-R	CACAAGATCAAAGTTTCCCTCAATCTCTTAGTCGACTCTAC
